# Type II and type IV toxin–antitoxin systems show different evolutionary patterns in the global *Klebsiella pneumoniae* population

**DOI:** 10.1093/nar/gkaa198

**Published:** 2020-03-31

**Authors:** Gal Horesh, Cinzia Fino, Alexander Harms, Matthew J Dorman, Leopold Parts, Kenn Gerdes, Eva Heinz, Nicholas R Thomson

**Affiliations:** 1 Wellcome Sanger Institute, Wellcome Genome Campus, Hinxton, Cambridgeshire, CB10 1RQ, UK; 2 Centre of Excellence for Bacterial Stress Response and Persistence, Department of Biology, University of Copenhagen, Copenhagen, 2200, Denmark; 3 Department of Computer Science, University of Tartu, Tartu, 50090, Estonia; 4 Department of Vector Biology, Liverpool School of Tropical Medicine, Liverpool, L3 5QA, UK; 5 Department of Infectious and Tropical Diseases, London School of Hygiene & Tropical Medicine, London, WC1E 7HT, UK

## Abstract

The *Klebsiella pneumoniae* species complex includes important opportunistic pathogens which have become public health priorities linked to major hospital outbreaks and the recent emergence of multidrug-resistant hypervirulent strains. Bacterial virulence and the spread of multidrug resistance have previously been linked to toxin–antitoxin (TA) systems. TA systems encode a toxin that disrupts essential cellular processes, and a cognate antitoxin which counteracts this activity. Whilst associated with the maintenance of plasmids, they also act in bacterial immunity and antibiotic tolerance. However, the evolutionary dynamics and distribution of TA systems in clinical pathogens are not well understood. Here, we present a comprehensive survey and description of the diversity of TA systems in 259 clinically relevant genomes of *K. pneumoniae*. We show that TA systems are highly prevalent with a median of 20 loci per strain. Importantly, these toxins differ substantially in their distribution patterns and in their range of cognate antitoxins. Classification along these properties suggests different roles of TA systems and highlights the association and co-evolution of toxins and antitoxins.

## INTRODUCTION

The *Klebsiella pneumoniae* species complex, which includes *K. pneumoniae sensu stricto*, *Klebsiella quasipneumoniae* and *Klebsiella variicola*, is a major threat to public health. Members of this species complex are leading causes of opportunistic infections in hospitalised or immunocompromised patients (1,2) and increasingly linked to major hospital outbreaks by highly multidrug resistant isolates (3,4). Of particular concern is the recent emergence of convergent multidrug-resistant and hypervirulent strains; these two phenotypes, both carried on mobile elements, were so far considered mutually exclusive. These new strains cause serious community-acquired infections in otherwise healthy individuals with few treatment options given their extensive drug resistance profiles (5–9).

The spread of genetic elements that act as the vectors of antibiotic resistance and virulence determinants have repeatedly been linked to toxin–antitoxin (TA) systems (10–12). TA systems were first discovered as loci that enforce the maintenance of plasmids via post-segregational killing (13). They are comprised of bicistronic operons encoding a toxin which inhibits cellular processes, such as translation or DNA replication and an antitoxin which counteracts the toxins’ harmful activity. Typically, the antitoxin is less stable than the toxin, and thus following binary fission the antitoxin degrades more rapidly leading to any plasmid-free daughter cell being killed by the more stable toxin.

Since their first description, it has become clear that TA systems are ubiquitous across a broad range of prokaryotic plasmids and chromosomes (14–19). Furthermore, in addition to post-segregational killing they have roles in other important cellular processes such as the formation of antibiotic-induced persistence (20), defence against bacteriophages, biofilm formation (21–23) and through transcriptional read-through can influence the expression of adjoining genes (24).

There are six different types of TA systems, defined by the antitoxin and its mode of inhibition of the toxin (16,22,25). The most well studied TA systems, and the focus of this study are the type II TA systems: both toxin and antitoxin are proteins with the antitoxin inhibiting the toxin's activity through direct interaction. Similarly, type IV TA systems, also included in this study, are comprised of protein toxin and antitoxin however, their antitoxin inhibits the toxin's activity by interacting with the toxin's target.

Whilst TA systems have been well studied in a limited number of laboratory and clinical isolates of *Escherichia coli* (20,26–28) and *Salmonella enterica sv*. Typhimurium (29) little is known about their distribution or their full diversity in the *K. pneumoniae* species complex (19,30). Furthermore, there have been no studies in any bacterium that have considered investigating these systems using large clinically relevant collections.

Here we present the detection and phenotypic testing of known, variant and novel TA combinations as well as a systematic analysis of the diversity of TA systems in a collection of 259 *K. pneumoniae* species complex strains, including *K. pneumoniae sensu stricto*, *K. quasipneumoniae* and *K. variicola* (31). Whilst TA systems have been known to be common in *K. pneumoniae* plasmids and chromosomes based on studies with a small number of isolates (15,19), we show that they differ substantially in their distribution patterns and in the nature of the pairings between toxins and cognate antitoxin. Moreover, some TA systems are associated with the presence of clinically important genes, others are ubiquitous or specific to a species within this complex, alluding to different evolutionary dynamics. This comprehensive analysis highlights the different evolutionary processes under which these genes are inherited, the fluid association and co-evolution of toxins and antitoxins and reveals the complexity of gene evolution in a bacterium with high rates of horizontal gene transfer.

## MATERIALS AND METHODS

### Strains and phylogenetic analysis

Assemblies of 259 *K. pneumoniae* species complex strains (Supplementary Table S1) were assembled using VELVET (v1.2.07) (32) and annotated using PROKKA (v1.5) (33,34). Prokka combines the use of five other tools to identify features in the assemblies; Aragorn (v2.36) for tRNAs (35), Prodigal (v2.6) for coding sequences (CDSs) (36), RNAmmer (v1.2) for rRNAs (37), Infernal (v1.1) for non-coding RNA (38) and SignalP (v4.1) for signal leader peptides (39). The core gene phylogeny was inferred from a core gene alignment generated using Roary (40), and a maximum likelihood tree from the informative SNPs, chosen using SNP-sites (41) (v2.3.2), was constructed using RAxML (v8.2.8) (42) with 100 bootstrap replicates.

### Toxin–antitoxin prediction

SLING (v1.1), a tool to identify linked genes (43), was used to search for toxins and their cognate antitoxins using the built-in toxin domain database provided in SLING. Briefly, SLING uses hidden markov models of known toxin domains to search the genomes for putative toxins. Following the toxin search, SLING will search for an adjacent CDS for the cognate antitoxin based on a set of given structural requirements. We applied the default structural parameters for a TA search in the filtering step (minimum toxin length: 30 aa, maximum toxin length: 200 aa, minimum antitoxin length: 50 aa, maximum antitoxin length: 150 aa, maximum overlap between toxin and antitoxin: 20 bp, maximum distance between toxin and antitoxin: 50 bp, order: antitoxin either upstream or downstream to toxin). SLING provides an expected length for some of the toxin domains. In that case, only CDSs which were no more than 100 aa longer or shorter than the expected length were accepted. Following the filtering, SLING uses pairwise protein sequence alignments to assign clusters to all toxins and antitoxins.

The local sequence identity and alignment coverage per toxin and antitoxin group was taken from the BLAST+ results. All the antitoxin and toxin sequences from each group were aligned using MUSCLE (v3.8.31) (44). The global sequence identity was calculated as the pairwise sequence identity between every two sequences in the multiple sequence alignment.

### Statistical analysis

Statistical analyses were performed in R (v3.3.1). Briefly, the toxin and antitoxin accumulation curves were generated using the *specaccum* function in the vegan (45) library with 100 random permutations. Principal component analysis was performed using the *prcomp* function. Association between toxins and lineage or the presence of antimicrobial resistance (AMR), virulence or plasmid replicons were performed using Fisher's exact test and corrected for multiple testing using the false discovery rate (FDR) with the *p.adjust* function. Differences between groups (*K. pneumoniae* species complex, toxin categories) were assessed using the Wilcoxon test and corrected using FDR.

### Toxin group classification

Toxin groups which were found in over 80% of isolates of all species were assigned as ‘ubiquitous’. Toxin groups which had at least four copies and were found to be significantly associated with *K. pneumoniae* complex species (Fisher's exact test, FDR corrected, *P* < 0.01) were assigned ‘species associated’. Toxin groups which were not ubiquitous or species associated were assigned ‘sporadic’ if they had 26 copies or more or otherwise, if they were found to be significantly associated with the presence of AMR genes, virulence genes or plasmid replicons (Fisher's exact test, FDR corrected, *P* < 0.01). The remaining toxin groups were assigned ‘rare’. Changing the sequence similarity thresholds for grouping toxins increased the number of toxin groups however the number of ubiquitous, species-associated and sporadic toxin groups stay constant (Supplementary Figure S1A). There is an increase in the number of rare toxin groups which is driven by an increase in the number of singleton toxin sequences. The ubiquitous toxin groups and species-associated toxin groups were robust and stable across all identity thresholds (Supplementary Figure S1B and C). Our chosen BLAST identity cutoff of 75% allows separation of sequences which share similar domains, for instance, DNA binding domains, yet keeps homologous sequences together and does not separate sequences by species due to drift (Supplementary Figure S1D and E).

### Definition of novel versus known antitoxins

All *in-silico* predicted and experimentally validated type II and IV antitoxin sequences were downloaded from the TA database TADB (v2, downloaded on 27.08.17) (46,47) and performed pairwise comparisons between all antitoxin sequences identified by SLING using protein-protein BLAST+ (v2.7) (48). A SLING antitoxin group was marked as ‘known’ if one or more of the antitoxins in that group shared at least 75% identity and an *E*-value of 0.01 or lower with an antitoxin from TADB (consistent with our definition of an antitoxin group). Interpro-scan (v5) was used to assign function to the sequences of the novel antitoxins (49). Sequences which were predicted to be antitoxins by Interpro-scan were also marked as ‘known’. Otherwise, the group was marked as ‘novel’.

### Orphan antitoxins

Antitoxin sequences from an antitoxin cluster were grouped using cd-hit (v4.7) (50) with an identity threshold of 90% and word size of five to remove redundant sequences. An antitoxin protein database of the cd-hit representative antitoxins was constructed using BLAST+ (v2.7) (48). The six frame-translated *K. pneumoniae* genomes from the SLING output (43) were aligned against the antitoxin database using blastn. A CDS was considered an ‘orphan antitoxin’ if (i) it was between 50 and 150 aa long, (ii) it shared 75% sequence identity or more to an antitoxin in the collection and (iii) the alignment was 50 aa or longer. These settings were chosen to be consistent with our definition of identity between antitoxin sequences in our original analysis. The sequences 1000 bp upstream and downstream to the orphan antitoxins were clustered with the respective 1000 bp sequences surrounding the original antitoxin in the viable TA pair using cd-hit-est with 80% identity threshold and word size of five. If orphan antitoxin context sequences were in the same cd-hit cluster as the sequences of the original antitoxin, they were marked as ‘same’ and ‘different’ otherwise.

### Identification of AMR genes, virulence genes and plasmid replicons

A collection AMR genes were obtained from the modified version of ARG-ANNOT available on the SRST2 website (https://github.com/katholt/srst2/tree/master/data, downloaded on 02.10.16) (51,52). A dataset of virulence factors was obtained from the *Klebsiella*-specific BIGSDB (http://bigsdb.pasteur.fr/klebsiella/klebsiella.html, downloaded on 22/02/16). The PlasmidFinder database (v1.3) of plasmid replicons was downloaded using ARIBA (v2.12) (53,54). Presence or absence of a gene in a genome was determined using ARIBA (v2.12) with default settings (54). Nucleotide–nucleotide BLAST+ (v2.7) of the VELVET assemblies against the target gene databases was used to identify contigs which contain a gene of interest (AMR, virulence or plasmid) (48). A match was determined if any of the associated genes had a BLAST bit score of 200 or more.

### Phenotypic testing

Bacterial strains, plasmids and oligonucleotides used in this study are listed in Supplementary Table S2. The sequences of synthesised genes, including mutated ribosomal binding sites and restriction sites where appropriate, are listed in Supplementary Tables S3 and 4.

Strains were cultured routinely on LB media. Where appropriate, bacteria harbouring plasmids were cultured on LB media supplemented with 100 μg/ml ampicillin or 30 μg/ml chloramphenicol.

Toxin and antitoxin sequences predicted from computational analysis were synthesised, cloned and sequence-verified using the GeneArt DNA synthesis service (ThermoFisher Scientific, DE). Toxin sequences were cloned into pNDM220 under P*lac* control (55), and antitoxin sequences into pBAD33 under P*ara* control (56) (Supplementary Tables S3 and 4). LB agar plates were supplemented with 1 mM of isopropyl β-D-thiogalactopyranoside (IPTG) for the induction of P*lac* and 0.2% w/v of L-arabinose for the induction of P*araB*. Overnight cultures were washed once and then serially diluted (10^−1^ to 10^−6^) in sterile phosphate-buffered saline (PBS). 10 μl of the original and diluted cultures (10^−1^ to 10^−6^) were spotted on LB agar plates containing the induction supplements.

Lyophilised plasmids were rehydrated in nuclease-free water. In order to ensure that *in vitro* validation experiments were performed using a single clone of each synthesised construct, each plasmid was propagated and prepared from a cloning strain of *E. coli*. Briefly, *E. coli* was cultured aerobically in 100 ml LB broth to an OD_600_ of ∼0.5 (200 rpm, 37°C). Cells were harvested by centrifugation and resuspended in ice-cold 10 mM calcium chloride (CaCl_2_) solution. Cells were washed three times in CaCl_2_ solution, collected by centrifugation, resuspended in 10 mM CaCl_2_ containing 25% v/v glycerol, and frozen at −80°C. One microlitre of each plasmid solution was used to transform these chemically competent *E. coli* by heat shock (plasmid incubated with bacteria on ice for 30 min, heat shock at 42°C for 30 s, 5 min immediate recovery on ice). Transformed cells were recovered for 1 h at 37°C (200 rpm), and transformants were selected for on solid LB media supplemented with appropriate antibiotics. One colony was picked and single-colony purified; the purified clone was then cultured overnight in 5 ml LB supplemented with antibiotics. Plasmids were extracted from 2 ml of each culture using the QIAprep Spin Miniprep kit (Qiagen, #27104) and the remaining culture was mixed with glycerol (25% v/v final concentration) and stored at −80°C.

## RESULTS

### Type II and type IV TA systems are highly abundant in the *K. pneumoniae* species complex

**Figure 1.**
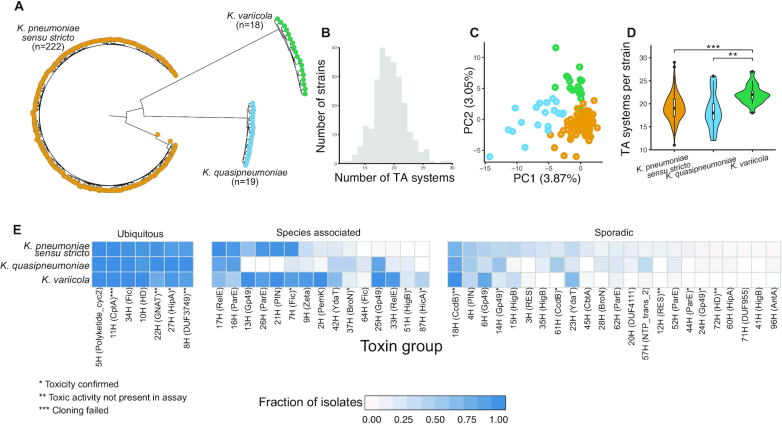
Diversity of toxins in *Klebsiella pneumoniae* species complex. (**A**) Core gene phylogeny of the 259 selected *K. pneumoniae* species complex genomes. (**B**) Number of predicted TA systems per isolate. (**C**) First two principal components of PCA analysis of toxin repertoire coloured by *K. pneumoniae* complex species (yellow: *K. pneumoniae sensu stricto*, blue: *Klebsiella quasipneumoniae*, green: *Klebsiella variicola)*. (**D**) Number of predicted TA systems per isolate, stratified by *K. pneumoniae* complex species. Pairwise Wilcoxon rank sum test, *P < 0.01, **P < 0.001, FDR corrected. (**E**) Fraction of isolates from each *K. pneumoniae* complex species possessing each of the toxin groups. Toxin groups are categorised by their distribution patterns (detailed in Supplementary Table S5). The toxin Pfam profile used to identify the toxin group is in brackets.

A total of 259 *K. pneumoniae* species complex genomes representing the global diversity were included in this study (31) (Supplementary Table S1). These include 222 *K. pneumoniae sensu stricto*, 18 *K. quasipneumoniae* and 19 *K. variicola* isolates (Figure 1A), including isolates taken from community and hospital acquired infections, those causing invasive and non-invasive disease and those isolated from both animals and plants (31). Although four additional species from this complex have been described (57–59), our study focuses on these three species as there is a well described dataset consisting of these species that reflects the clinically relevant diversity of the *K. pneumoniae* species complex (31).

SLING was used to search for TA pairs within our genomic dataset (43). SLING uses Pfam hidden Markov model profiles of known toxins to search for candidate toxins within a given genomic dataset using HMMER (60,61). Where identified, SLING searches for cognate protein-coding antitoxins in proximity to the identified toxin, following a set of predefined structural rules. We applied conservative requirements for a TA pairing to be considered a valid TA system and discarded any putative toxins and antitoxins which deviated from our criteria (see ‘Materials and Methods’ section). Finally, all candidate toxin and antitoxin pairs are grouped according to sequence similarity using a cut off of 75% local amino-acid sequence identity. For clarity, a group of toxins or antitoxins which have been clustered together based on their amino-acid sequence identity are referred to as ‘toxin group’ and ‘antitoxin group’, respectively. The toxin groups are named by the profile by which they were found.

Using a collection of 55 (52 type II, 3 type IV) Pfam toxin profiles as our input for the search strategy (43), we identified a total of 140 toxin groups (130 type II, 10 type IV) and 233 antitoxin groups (211 type II, 23 type IV), forming 244 different TA structures in the genomes included in this study (Supplementary Tables S5 and 6). Altogether, TA systems were highly prevalent in all members of the *K. pneumoniae* species complex, with a median of 19 loci per isolate genome (range 11–29, Figure 1B). Principal component analysis showed a clear separation into the three species based on toxin repertoire (Figure 1C). Furthermore, *K. variicola* has a higher median of 22 TA systems per isolate compared to 18 and 19 in the other two species (Figure 1D; pairwise Wilcoxon rank sum test *P* < 0.01, FDR corrected).

Based on sequence similarity, the number of defined toxin groups per toxin Pfam profile ranges from 1–13 (see Supplementary Table S7). The mean sequence variation within any one toxin group ranged from 68.95 to 100% local identity at the amino-acid level covering 59.33–100% of the full length of the protein (46.37–100% amino-acid identity over the complete protein) (Supplementary Table S5). This highlights the diversity of candidate toxins linked to functionally tested domains that were identified in this study. For instance, we aligned the sequences of a toxin group 31H containing the HicA domain to the toxins containing the HicA domain taken from the existing TA database, TADB (46,47) (Supplementary Figure S2). Whilst some key residues are conserved throughout, there are considerable variations between the sequences taken from TADB to each other as well as to our predicted toxin.

### Redefining toxins based on their distribution patterns

We classified the 140 identified toxin groups into four categories based on their distribution in our dataset (see ‘Materials and Methods’ section) (Figure 1E; Supplementary Figure S3 and Table S5). Seven toxin groups were ubiquitous (one type IV), present in over 80% of the isolates included in this study and from all three species. Fifteen toxin groups, all type II toxins, differed in prevalence between the three species (Fisher's exact test *P* < 0.01, FDR corrected, Figure 1E). Twenty-three toxin groups (one type IV) (17%) were distributed sporadically with no species association, including a number which were associated with clinically relevant genes. Finally, the remaining 95 toxin groups (eight type IV) (68%) were rare and found in fewer than 10% of the isolates (Supplementary Figure S3).

Within the ubiquitous toxin groups, we observed significantly higher nucleotide identity for toxins within the same species compared to toxins from other species (median 99.4% compared to 93.51%, Wilcoxon rank sum test, *P* < 0.001, Supplementary Figure S4). The median nucleotide identity for sporadic toxin groups for toxins within a species was 97.06% compared to 96.57% between species. This elucidates the evolution of the ubiquitous toxin groups due to genetic drift within a specific member of the species complex, compared to the likely mobile, sporadic toxin groups where this effect is not observed.

The seven ubiquitous toxin groups are known to inhibit translation via mechanisms that do not include RNA cleavage: toxin group 5H (polyketide_cyc) is a homolog of the RatA toxin in *E. coli* which inhibits translation by binding to the 50S ribosomal subunit (62). Similarly, toxin group 34H (Fic) is a Doc toxin which inhibits translation by phosphorylating and concomitantly inactivating elongation factor TU (EF-Tu) (63). Toxin groups 22H and 8H with the GNAT and DUF3749 domains are acetyltransferases known to inhibit translation by acetylating aminoacyl-tRNA (30,64). Group 27H contains a HipA domain which is well described for its association with the high persister phenotype (20,65) and inhibits translation by phosphorylating and concomitantly inactivating glutamyl-tRNA synthetase (66). Toxin group 11H with the CptA domain belongs to type IV TA system which inhibits cytoskeleton assembly (67). Finally, group 10H with the HD domain is a phosphohydrolase which is a putative toxin domain from TADB but its exact function is unknown (43,46–47).

The species associated toxin groups present different distribution patterns across the three *Klebsiella* species used in this study. *Klebsiella pneumoniae sensu stricto* possesses three toxin groups in lower prevalence compared to the other two species (51H (HigB), 64H(Fic) and 25H (Gp49)) (Figure 1E). *Klebsiella variicola* possess five toxin groups in higher prevalence compared to *K. pneumoniae sensu stricto* and *K. quasipneumoniae* (42H (YdaT), 9H (Zeta), 2H (PemK), 33H (RelE) and 87H (HicA) domains). Toxin group 87H (HicA) is specific to *K. variicola* and is not observed in the other two species in our dataset. On the other hand, toxin groups 16H (ParE) and 17H (RelE) domains are less common in *K. variicola*. Finally, *K. quasipneumoniae* lacks three toxin groups (21H (PIN), 26H (ParE) and 13H (Gp49)), and rarely possesses toxin group 7H (Fic). On the other hand, toxin group 37H (BroN) is observed in higher prevalence in *K. quasipneumoniae* relative to the other two species. Of these *K. quasipneumoniae* isolates, 11% possess three copies of this toxin group and 16% possess two copies (Supplementary Figure S5).

### Prediction of novel antitoxins

**Figure 2.**
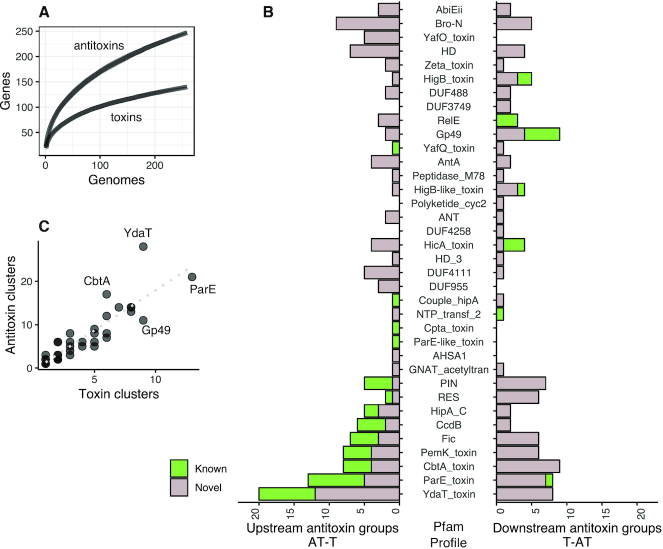
Identification of novel antitoxins in the *Klebsiella pneumoniae* genomes. (**A**) Accumulation curves of unique toxins and antitoxins groups found in an increasing collection of *K. pneumoniae* genomes. (**B**) Count of antitoxin groups found only upstream (AT-T) and downstream (T-AT) relative to each toxin Pfam profile, coloured by known or novel. (**C**) Number of toxin groups of each toxin Pfam profile, relative to the number of antitoxin groups found in their proximity.

Accumulation curves of the unique toxin and antitoxin groups identified using SLING suggest that sampling additional *K. pneumoniae* species complex genomes will lead to further identification of new candidate antitoxins (Figure 2A) (43). To assess whether the identified antitoxins were known or novel, we compared their sequences to all type II and type IV antitoxin sequences retrieved from the TADB database (46,47) (see ‘Materials and Methods’ section). 195 (173 type II, 22 type IV) of the 233 (211 type II, 23 type IV) antitoxins detected in our study were not found in TADB and seen to be novel candidate antitoxins linked to a known toxin (Supplementary Table S6). For completeness, we assigned function to the 195 novel antitoxin groups using interpro-scan (Supplementary Table S6) (49). We identified 19 additional antitoxin groups which matched known antitoxins by interpro-scan which were not in TADB (antitoxins of toxin profiles YdaT (8), CbtA (4), CcdB (2), Fic (1), PemK (1), PIN (1), HigB (1) and HicA (1)), leading to a final count of 176 novel antitoxins (76%).

About 72% of novel antitoxins (127/176) could not be assigned a putative function (Supplementary Table S6). Five groups contain one of the toxin profiles used in the toxin search and are the result of disrupted toxins. Twelve groups were predicted to be DNA binding or transcriptional regulators which are plausible functions for antitoxins due to the auto-regulation of the TA operon through conditional cooperativity (22,68). Another 12 groups were assigned to be intrinsically disordered proteins (69). The remaining groups contain profiles indicating other functions, such as domains of unknown function, ABC transporters, prophages and other functional categories (Supplementary Table S6).

For each of the toxin groups, we examined the arrangement of the linked antitoxin: upstream of the toxin (denoted AT-T) or downstream of it (denoted T-AT) (Figure 2B). Of the known antitoxins, 72% were located upstream of the toxin compared to 50% of the novel antitoxins (*P* = 0.007, Chi squared test).

Looking at the association between specific toxins and antitoxins we found that with a greater number and diversity of defined toxin groups belonging to the Pfam profile used to search for the toxins, there were concomitantly more antitoxin groups linked to those toxins (0.88 Pearson correlation, Figure 2C). The exceptions include the YdaT domain which was found with 28 candidate antitoxin groups and linked to only 9 toxin groups. This both suggests there is coevolution of TA pairs along with instances where a range of different antitoxins can inhibit the same toxin.

### Fluid association and distribution of toxin–antitoxin pairings

We found that a single toxin group can be found with up to a maximum of 12 discrete antitoxins, highlighting the ‘mix and match’ nature of TA associations (70). It is important to note that the antitoxin groups are substantially different from each other as we applied a cut off of 75% local amino-acid sequence identity for two antitoxins to be in the same group. Furthermore, the mean sequence variation within any one antitoxin group ranged from 74.64–100% local identity at the amino-acid level covering 61–100% of the alignment length (59.88–100% aa identity over the complete protein), highlighting further the diversity in the candidate antitoxins identified (Supplementary Table S6).

**Figure 3.**
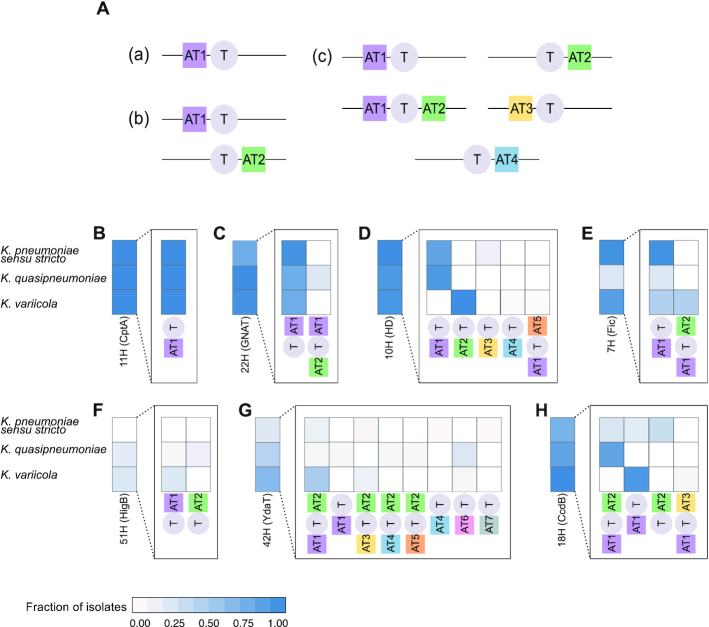
Diversity in the observed operon structures for the different toxin categories. (**A**) Examples of range of antitoxins and possible operon structures for a toxin (a) toxin group found in a single structure with a single antitoxin group (b) toxin group found in two different structures with two different antitoxin groups (c) toxin group found in five different structures with four different antitoxin groups. (**B**–**H**) Fraction of isolates from each *Klebsiella pneumoniae* species complex possessing each of the operon structures of seven example toxin groups: (B–D) ubiquitous, (E and F) species associated, (H) sporadically distributed.

In addition to a range of different antitoxins paired to the same toxin, toxins also showed a range of operon structures (Figure 3A); some toxin groups were linked to a single antitoxin in a conserved position either upstream or downstream of the toxin. Other toxin groups were found in multiple arrangements with the antitoxin sequence and/or location of the antitoxin relative to the toxin changing (Figure 3B–H). For the ubiquitous toxin groups, only three groups were found in a single arrangement (groups 11H (CptA, a type IV toxin), 5H (polyketide_cyc) and 8H (DUF3749)) (Figure 3B and Supplementary Figure S6). Three other toxin groups (groups 22H (GNAT), 34H(Fic) and 27H(HipA)) were observed in two or three structures often with one structure dominating (>90% of isolates) and the others being rare occurrences of the other structures (<3% of isolates, Figure 3C and D). Although the HD toxin group is ubiquitous, one TA arrangement, found in 80% of isolates, is specific for *K. pneumoniae sensu stricto*, missing in *K. variicola* and replaced by a structure specific to *K. variicola* (Figure 3D and Supplementary Figure S6).

The species-associated toxin group 7H (Fic), is found in one arrangement which is specific to *K. variicola* (Figure 3E). Toxin group 51H (HigB) is associated with two unique antitoxins with one being specific to *K. quasipneumoniae* (Figure 3F). Alternatively, other toxin groups possess multiple operon structures with no clear species association, for instance, toxin group 42H (YdaT) is observed with seven antitoxin groups in eight different arrangements (Figure 3G and Supplementary Figure S7). Other than in a single case (18H (CcdB)), the sporadically distributed toxins were not seen in species-specific arrangements emphasising they are unlikely to be vertically inherited (Figure 3H and Supplementary Figure S8)

Most of the antitoxins we identified were toxin group specific. However, antitoxin from group 52P was found both with toxin group 31H (HicA) in seven isolates and with toxin group 54H (BroN) in a single isolate. Interestingly, it was always found upstream to the 31H (HicA) toxin and downstream 54H (BroN) toxin. The antitoxin proximate to 31H (HicA) shares 83.2% amino acid sequence identity with the antitoxin proximate to 54H (BroN) antitoxin. This antitoxin is not found in TADB but encodes for a domain of unknown function DUF1902 (PF08972) which is in the same Pfam clan as many other antitoxins (Met_repress, CL0057).

### Phenotypic testing *in silico* predictions of toxins and confirmation of novel antitoxins

Due to the apparent diversity of TA systems within and between species and the novel combinations of toxin and antitoxins found in this study, we tested the ability of 17 candidate toxins, representing the diversity of toxins within a given group and from a range of genomic backgrounds, to inhibit bacterial growth in our *E. coli* model system (see ‘Materials and Methods’ section). Selected were: four ubiquitous, four species associated, seven sporadically distributed and two rare candidate toxins (Supplementary Table S3 and Figure S9).

We confirmed the toxicity of all the species associated toxins that we tested (groups 51H (HigB), 7H (Fic), 87H (HicA) and 37H (BroN)). Of the remaining toxins we were able to observe toxicity from the 27H (HipA) toxin group which is ubiquitous across the species complex as well as four of the seven sporadically distributed toxins tested from groups 14H (Gp49), 24H (Gp49), 61H (CcdB), 44H (ParE) and a rare toxin from the 31H (HicA) group. The ubiquitous type IV toxin we tested, 11H ((CptA)), could not be successfully synthesised or cloned, likely due to its toxic activity. The rest of the toxins tested showed no toxic activity under the conditions tested in our assay (summarised in Supplementary Table S3).

**Figure 4.**
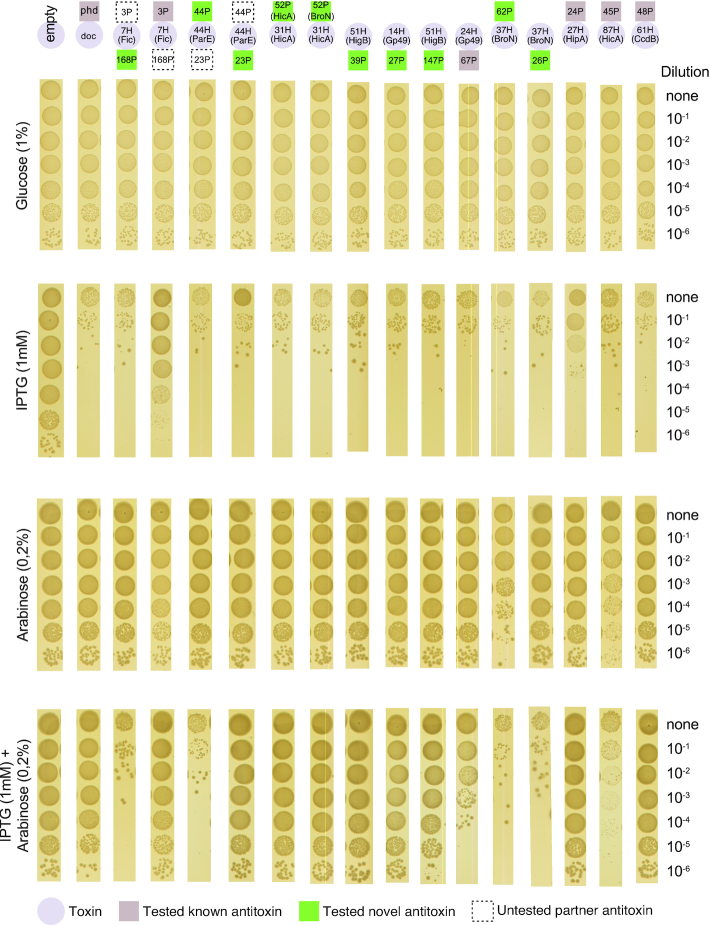
Phenotypic testing predicted TA combinations. Toxins in circles, tested novel antitoxins in green and tested known antitoxins in grey. For operon structures AT1-T-AT2, the untested partner antitoxin is in a dashed square. LB agar plates were supplemented with 1 mM IPTG for the induction of toxin P*lac* promoters and 0.2% w/v of L-arabinose for the induction of antitoxin P*ara* promoters. Overnight cultures were serially diluted (10^−1^ to 10^−6^) in PBS. Figures were cropped from the original images for clarity. Original images available at: https://github.com/ghoresh11/kpneumoniae_TAs/tree/master/results/Functional_validation_orig.

Subsequently, we tested 14 candidate antitoxins for their ability to counteract the toxicity of their cognate toxin in our *E. coli* model system (including 10 novel antitoxins; this study; Figure 4 and Supplementary Table S4). Eight of the fourteen antitoxins (57%) led to complete inhibition of the toxic activity, five of which were novel antitoxins (summarised in Supplementary Table S4). Three of the confirmed novel antitoxins were predicted to contain DNA binding domains by interpro-scan (39P, 27P, 147P). One antitoxin contains a domain of unknown function (52P) and the final antitoxin did not match any existing entry in Interpro (44P). Three of the confirmed antitoxins in the T-AT format were located downstream of the toxin (groups 27P (Gp49), 147P (HigB) and 39P (HigB)). An additional known antitoxin only partially inhibited toxicity (67P).

For completeness, for operons that had the structure AT1-T-AT2, we tested both AT1 and AT2. In both cases, AT1 only was confirmed to inhibit the toxin's activity whilst we did not observe toxin inhibition activity with AT2.

Finally, these data also revealed some more unexpected findings. In two cases the predicted antitoxins were themselves found to be toxic in our experimental system (45P, 62P) (Figure 4). One of these antitoxins is a well-described antitoxin with a HicB domain (62P). In addition, we confirmed both versions of antitoxin group 52P, associated with toxins from markedly different groups (31H (HicA) and 54H (BroN)), were able to counter toxin group 31H (Figure 4; Supplementary Table S4 and Figure S9). Although the antitoxin group is linked to two different toxins and the two versions of the antitoxin share only 83.2% amino acid identity, both versions inhibit the activity of this toxin. We were unable to confirm the toxicity of toxin group 54H (BroN) (Supplementary Table S3 and Figure S9), thus we could not confirm inhibition of this toxin group. Finally, we tested two variants of the toxin group 51H (HigB); a shorter protein (53 aa) which was observed with antitoxin group 39P and a longer protein (103aa) observed with antitoxin group 147P. The C-terminus of the longer toxins is 83% identical to the shorter protein. The two antitoxins share 71% amino-acid identity. We were only able to confirm the toxicity of the shorter 51H toxin. Nonetheless, we tested both antitoxins 39P and 147P with the shorter 51H toxin, and found that both antitoxins are functional and able to inhibit the toxin.

### Orphan antitoxins are abundant in the population

**Figure 5.**
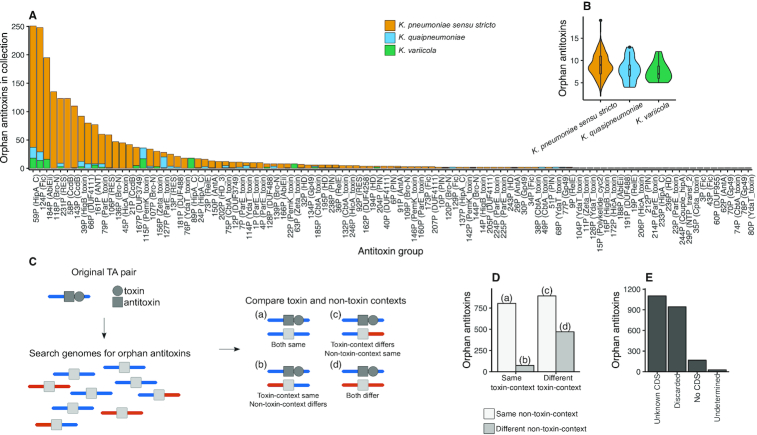
Orphan antitoxins in *Klebsiella pneumoniae* genomes. (**A**) Number of orphan antitoxins identified from each antitoxin group, coloured by *K. pneumoniae* complex species. The toxin Pfam profile of the toxin of the valid TA pair is in brackets. (**B**) Orphan antitoxins per strain stratified by *K. pneumoniae* complex species. (**C**) Illustration of context analysis applied to each orphan antitoxin. The flanking sequences around each orphan antitoxin were compared to the flanking sequences of the valid TA pair. Each flank was classified according to whether or not it matched the sequence of the original TA pair. (**D**) Number of occurrences of orphan antitoxins classified by the similarity of their contexts’ to the valid TA pairs’. (**E**) Presence of a CDS in the orphan antitoxin's toxin-context.

We sought to determine whether the antitoxins in the TA pairs were also present on the *K. pneumoniae* species complex genomes as orphan genes uncoupled to a candidate toxin gene. We aligned the predicted antitoxin sequences against all the genomes and found a total of 2253 occurrences of orphan antitoxins belonging to 105 of the 233 antitoxin groups defined in this study (96 type II and 9 type IV) (Figure 5A and Supplementary Table S8). Of these, 25% are known antitoxins found in TADB or Interpro (26/105). For 80% (77/96) of type II and 89% (8/9) of type IV antitoxin groups, we found fewer than 26 orphan copies in the entire genome collection, i.e. occurrences of unpaired antitoxins were rare and were found in fewer than 10% of genomes (Figure 5A). Conversely two antitoxin groups, containing the type II Fic and HipA toxin domains, were found unpaired in more than 80% of the genomes (>207 copies) across the species complex. In 35 of the 105 orphan antitoxin groups, we detected orphans in a species different to that of the valid TA pair (Supplementary Table S8). For instance, antitoxin group 89P of the HipA toxin was originally identified in *K. quasipneumoniae*. However, orphan antitoxins were found only in *K. variicola* (Figure 5A). Similarly, antitoxin group 115P belonging to a PemK-containing toxin was originally identified in *K. variicola*, but orphan antitoxins were found in *K. quasipneumoniae* as well. Altogether we did not observe significant differences in the number of orphan antitoxins per strain between the three species, with a median of nine orphans per strain across the three species (Figure 5B) (pairwise Wilcoxon rank sum test, FDR corrected, *P* > 0.05).

To assess the origin of orphan antitoxins we aligned the upstream and downstream sequence surrounding the antitoxin with those found in valid TA pairs (Figure 5C) (see ‘Materials and Methods’ section). About 39% of the orphan antitoxins (879/2253) share the same toxin-context as the valid TA pair. Of these, 92% also share the same non-toxin-context, indicating that they are in the same genetic context as the valid TA pairs from the same group (Figure 5D). 65% of orphans which did not share the toxin-context of the original TA pair (893/1374) do share the non-toxin context. In 20% of cases (470/2253) neither the toxin-context or the non-toxin-context match the valid TA pair, i.e. the orphan antitoxins were surrounded up- and downstream by unrelated sequences to any of our detected TA pairs.

To confirm whether these were truly orphan antitoxins, we searched for a CDS within the toxin-context that could function as the toxin. In 49% of orphans (1,107/2253) we found a CDS within the context region that does not contain a known toxin domain and could be a candidate for a novel toxin (Figure 5E). In 43% of cases (947/2253) a toxin containing the original Pfam profile used in the search was found but the CDS was discarded due to the conservative structural requirements we applied for a TA system (Figure 5E). These may be false negatives in our original analysis, or otherwise TAs which have diverged from the expected structure for a functional TA pair. In 8% of cases (171/2253) the predicted antitoxin was truly orphan as we could not find a CDS longer than 50 aa in the context region that could function as a toxin. In 1% of cases (28/2253), the orphan antitoxin was close to the contig edge or proximate to a region with more than eight unknown nucleotides (N/X) and therefore we could not confirm the presence or absence of a toxin in its proximity.

### The association between toxins and antimicrobial resistance genes, virulence genes or plasmid replicons

**Figure 6.**
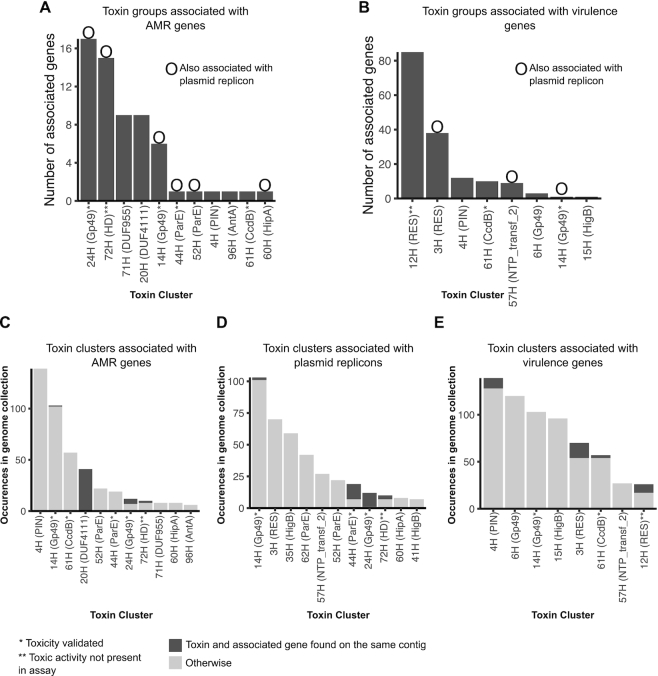
Toxin groups associated with AMR genes, virulence genes and plasmid replicons. Number of unique AMR (**A**) and virulence (**B**) genes associated with each of the toxin groups. Circles above bars indicate the toxin group is also associated with the presence of a plasmid replicon. Number of occurrences of toxins in the genome collection, for the toxin groups associated with AMR genes (**C**), plasmid replicons (**D**) and virulence genes (**E**). An occurrence of a toxin is coloured in dark if it is found on the same contig with one or more of the associated genes, light otherwise.

Considering the breadth of genomes included in this study we looked for physical linkage or co-occurrence between identified TA system genes and marker genes associated with horizontal gene transfer, AMR or virulence. Several of the sporadically distributed toxin groups were associated with clinically relevant AMR or virulence genes as well as plasmid replicons linked to the spread of AMR in *Klebsiella* and *E. coli* (Figure 6A and B, Fisher's exact test *P* < 0.01, FDR corrected). These included 24H (Gp49) and 72H (HD) toxin groups which were significantly associated with multiple AMR genes, including those conferring resistance to aminoglycoside, amphenicol, sulfonamide, tetracycline and beta-lactams, with 13–29% of toxin genes found on the same contig as the respective AMR genes (Figure 6C). 100% and 30% of toxins CDSs of toxin groups 24H and 72H, respectively, were on the same contig with an IncA/C plasmid replicon (Figure 6D). These contigs shared 99% (24H) and 97% (72H) sequence identity with the *K. pneumoniae* IncA/C-LS6 plasmid (JX442976), originally isolated from carbapenem-resistant *K. pneumoniae* (71), as well as AMR plasmids pNDM-KN (24H), pRMH760, pIMP-PH114 and pR55 (72H) (Supplementary Table S9) (72–75). Two toxin groups with a RES domain, 3H and 12H, were associated with multiple virulence genes (Figure 6B, Fisher's exact test *P* < 0.01, FDR corrected) and one of these groups (3H) with the presence of an IncHI1B plasmid replicon. Contigs containing these two toxins showed over 99% sequence identity to *K. pneumoniae* virulence plasmids pK2044 and pLVPK (Supplementary Table S9) (76,77). Five other toxin groups which were associated with AMR or virulence genes were also associated with the presence of plasmid replicons (Fisher's exact test *P* < 0.01, FDR corrected) (see Figures 6A–C).

## DISCUSSION

We present a systematic in-depth analysis of the diversity and evolution of TA systems in a large collection of a clinically important member of the *Enterobacteriaceae*, the *K. pneumoniae* species complex. We show that TA systems are highly prevalent in the species complex, however, the underlying processes of the evolution of TA systems are likely to be context-dependent. The toxins of these TA systems can be classified based on their distribution patterns as ubiquitous, species associated, sporadically distributed (often with associations to clinically important genes) or rare. The evolution of ubiquitous toxins is likely vertically inherited, as we observe higher nucleotide identity between toxins of the same species than between species. We do not observe the same effect for the sporadic toxins, suggesting that some TA systems are more mobile than others. Importantly, the classification presented in this study is based on the dataset used, which was aimed to capture the diversity of the *K. pneumoniae* species complex. It is possible that further sampling of under-represented lineages would increase power and refine the classification.

The pairing of antitoxin to toxin is not fixed; for each toxin we found a range of candidate antitoxins in different arrangements, putatively able to inhibit the same toxin. Sampling of more genomes lead to a large diversity in antitoxins relative to toxins, suggesting the potential number of interactions between toxins and antitoxins is large. Notably, some toxins are more stably coupled to a single antitoxin and observed in a single arrangement, whilst other toxins were observed with a wide range of antitoxins and operon arrangements. This highlights that the co-evolution between toxin and antitoxin is dependent on the system and context. This has functional implications as the antitoxin and its interaction with the toxin can affect the functioning of the TA system (78). Some antitoxins play a role in the regulation of the TA module as the TA pair regulate the expression of the TA operon (22,68). Furthermore, the interaction of the toxin with the antitoxin will determine the specificity of the inhibition and therefore would affect the dynamics of both activation and deactivation of the TA operon. Finally, antitoxin instability is often the result of degradation by proteases (22), therefore the inhibition of an antitoxin in response to stress can depend on the antitoxin sequence as it would determine the specificity of interaction with proteins that lead to its degradation (79). Future studies should be held to explore the biological implications of pairing different antitoxin groups with the same toxin group.

Even more, we often observe toxin or antitoxin groups which are specific to a species, i.e. a TA pairing is observed only in one particular genetic background. This suggests it may be beneficial to possess a specific TA pair under one genetic background compared to another. These results set the ground for future studies understanding the biological significance and expression of TA systems which are differentially distributed under different genetic backgrounds.

Altogether 76% of the identified candidate antitoxins we found were novel and not in the existing TA database TADB or Interpro (46–47,80). Furthermore, there is additional sequence diversity within each antitoxin group that we found. These results emphasise the potential large diversity of antitoxins that could inhibit these toxins and our lack of knowledge of the complete range and diversity of these systems.

Using an *E. coli* model system, we were able to confirm the toxicity of 10 of 17 tested toxins (∼59%) and the inhibitory activity of 10 of 14 tested antitoxins (∼71%). Nine of the tested antitoxins are novel and we were able to confirm the inhibition of five of them. We also found candidate antitoxins downstream of the toxin, and confirmed the inhibitory activity of three of them, highlighting exceptions to the common setup in which the antitoxin is encoded upstream of the toxin. These results could form the basis of future studies investigating how different autoregulatory principles enabled by upstream or downstream antitoxins might affect the biology of a TA system. Whilst some of these candidate antitoxins could be false predictions, the observation of known or confirmed antitoxins both upstream and downstream to toxins suggests we cannot rule out any antitoxin candidate. Importantly, a negative result in our assays does not rule out toxic or inhibitory activity of these proteins, but rather could be the result of confounding effects in our assays for example biological differences between *E. coli* K-12 and *K. pneumoniae*, lack of protein expression or incorrect folding in the heterologous host. Furthermore, our assays do not indicate whether these systems are expressed in the host bacterium or whether they have a physiological role in the host cell.

There is an abundance of orphan antitoxins present in the population which are unpaired to a functional toxin. These include a number of the antitoxins we expressed and were able to confirm their inhibitory activity (92 orphan copies of 39P, 17 orphan copies of 24P and 45 orphan copies of 45P, Figure 4, Supplementary Table S7). Sources of orphan antitoxins may be degrading TA pairs that are in different genetic locations, older degraded TA systems or otherwise, these could be candidates for new toxins which share the same antitoxin as we have identified. Alternatively, some orphan antitoxins may be paired to a known toxin but were discarded in our analysis due to the conservative structural criteria we defined for a TA system, suggesting that the prevalence of TA system in the *K. pneumoniae* species complex presented here may be underestimated.

These orphan antitoxins may be serving a new purpose. For example, they may serve as anti-addiction modules, preventing the fixation of plasmids or other mobile genetic elements (81). They may be interacting with the toxins of active TA pairs and affecting their function. Alternatively, they could also be conserved as remnants of a degraded TA locus that have acquired functions in transcriptional regulation of other genes in the genome (82).

The importance of this type of analysis is not limited to TA systems, and presents general trends to distinguish between groups of genes of other gene systems. Pan-genome analysis of bacterial datasets is often focused on the description of core compared to accessory genes without focussing on the precise details within these two categories. Here we showed that a more refined description of genes based on their distribution across the *K. pneumoniae* population and in the context of linkage to other genes. This finer grained analysis can be applied in other settings and lead to novel, highly relevant insights on evolutionary dynamics of poorly understood genetic elements.

## DATA AVAILABILITY

All scripts, files and original images present in this study are available at https://github.com/ghoresh11/kpneumoniae_TAs.

The accession numbers of all strains used in this paper are available in the Supplementary Tables S1.

## Supplementary Material

gkaa198_Supplemental_Files

## Notes

Present address: Alexander Harms, Biozentrum, University of Basel, Basel, Switzerland.
