# Supplementary material for: Type II and type IV toxin–antitoxin systems show different evolutionary patterns in the global Klebsiella pneumoniae population
Source: Nucleic Acids Res. 2020 Mar 31;48(8):4357–70. doi: 10.1093/nar/gkaa198 (PMC7192599; doi:10.1093/nar/gkaa198)
Supplement: gkaa198_Supplemental_Files [file gkaa198_supplemental_files.zip › supp_figures_new.pdf]

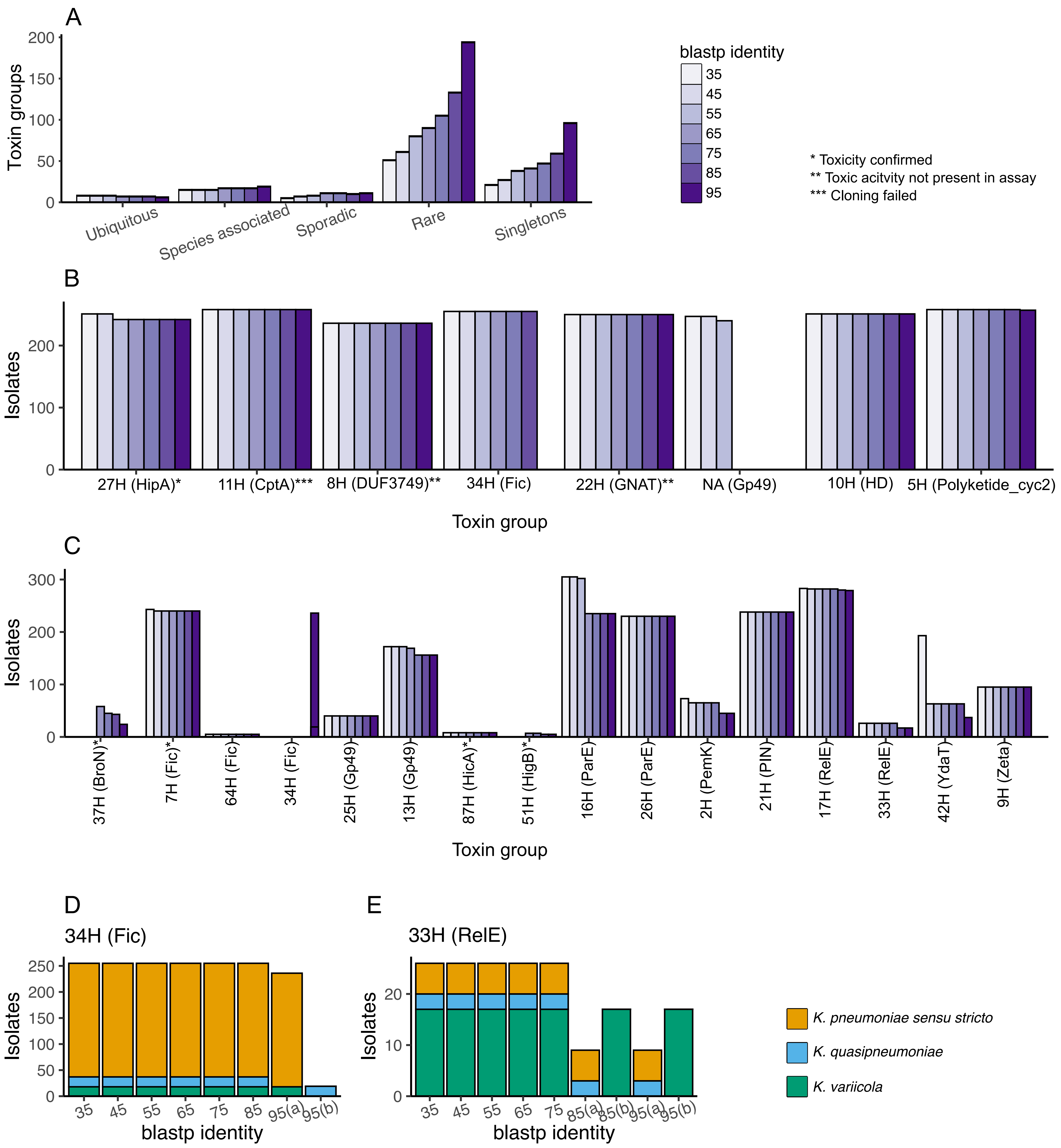

Figure S1: Effect of modifying blastp identity threshold on toxin clustering. **A** Number of toxin groups from each class per identity threshold. Singletons are toxin groups with only one member. Ubiquitous (**B**) and species associated (**C**) toxins per identity threshold. When bar missing, the toxin group was not ubiquitous or species associated for given threshold. Examples of clustering across thresholda for a ubiquitous toxin group Fic (**D**) and a species associated toxin groun RelE_1 (**E**).

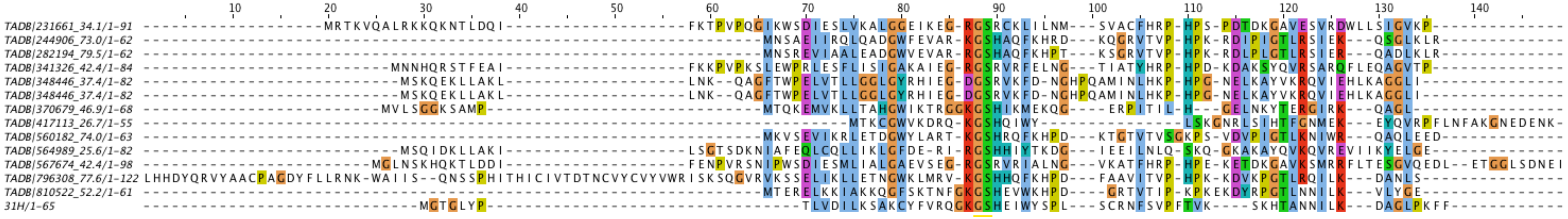

Figure S2: Multiple sequence alignment of all toxins sequences from TADB containing the HicA domain, and a representative of toxin group 31H containing the HicA domain in our dataset. Alignment produced using mafft (v7.205) (Katoh and Standley 2013). Image produced using JalView (v.210) (Waterhouse et al. 2009)

Figure S3: Distribution of toxins in K. pneumoniae. Toxins along the x-axis labelled according to SLING output. Coloured square indicates presence of toxin, white square absence. Toxins grouped according to distribution patterns. From left to right: ubiquitous (blue), species associates (green), sporadic with associations (red), sporadic with no associations (pink) and rare (orange). See Supplementary Table S2 for toxin label details.

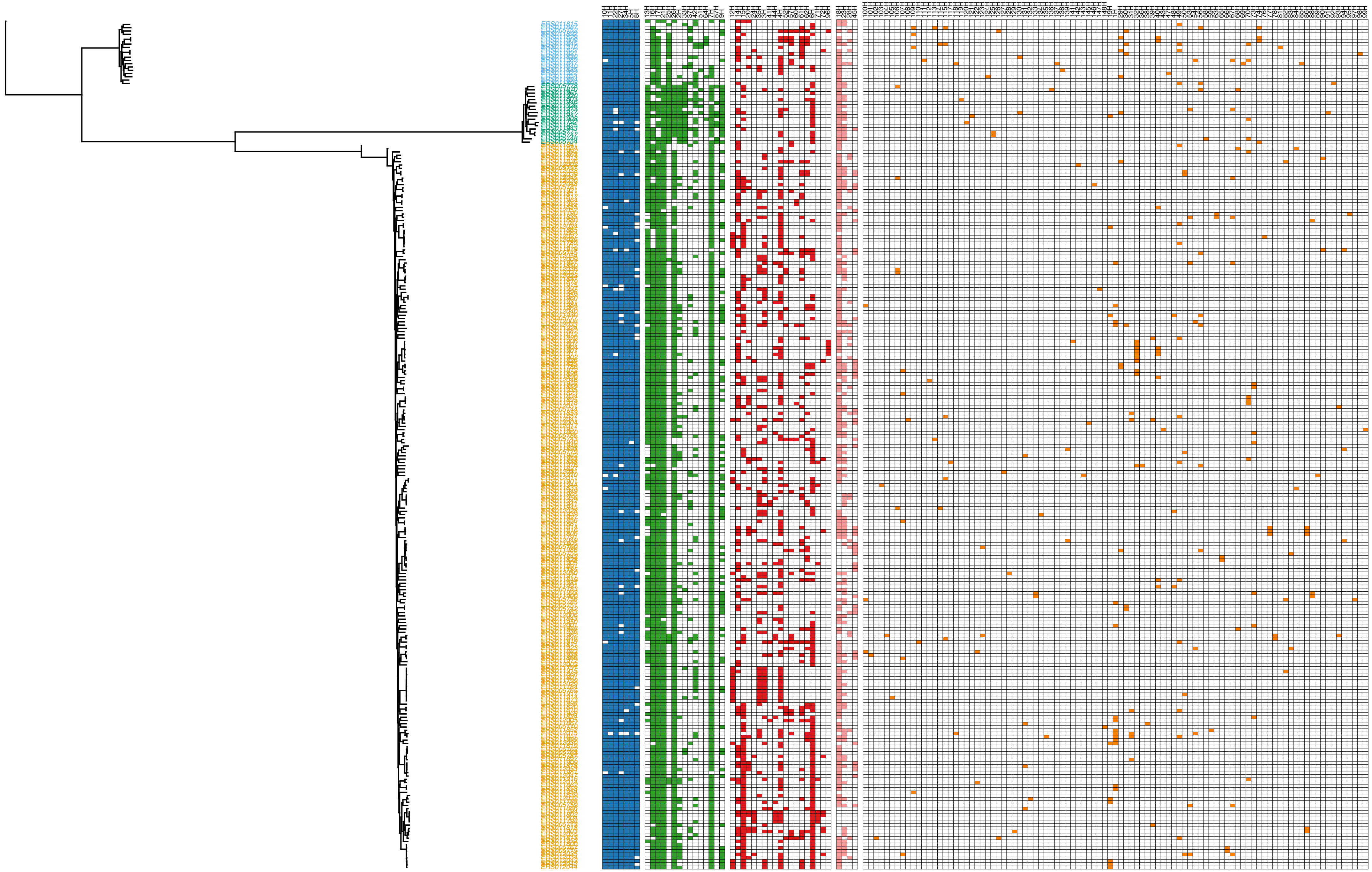

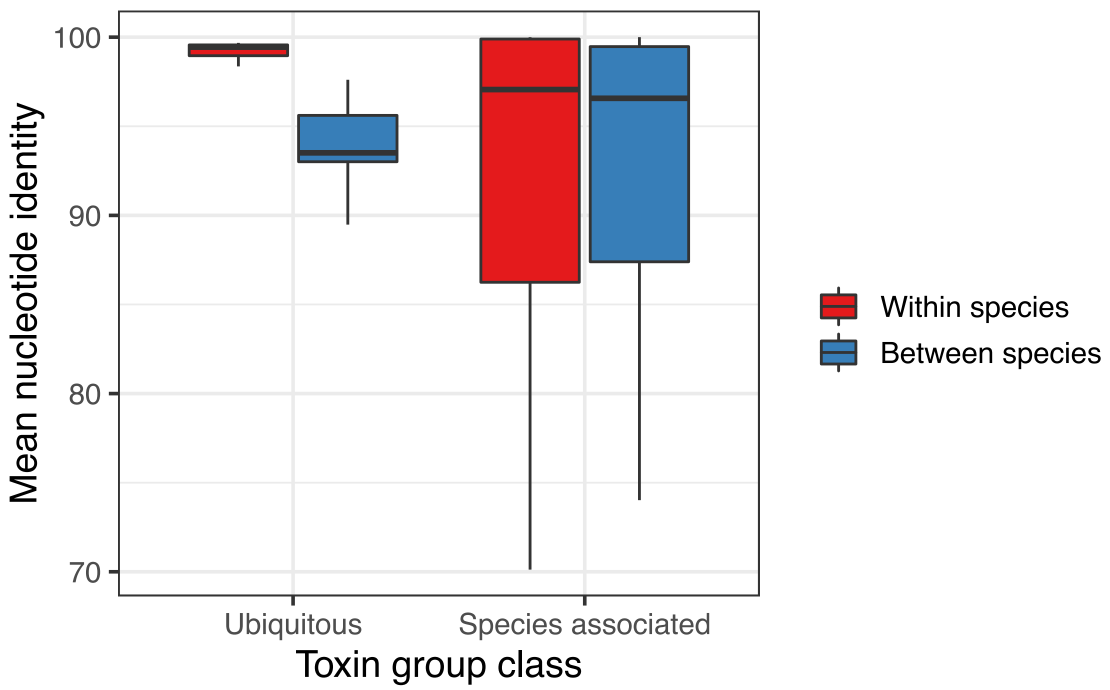

Figure S4: Mean nucleotide identity between toxins originating from the same species and different species for all ubiquitous and sporadic toxin groups

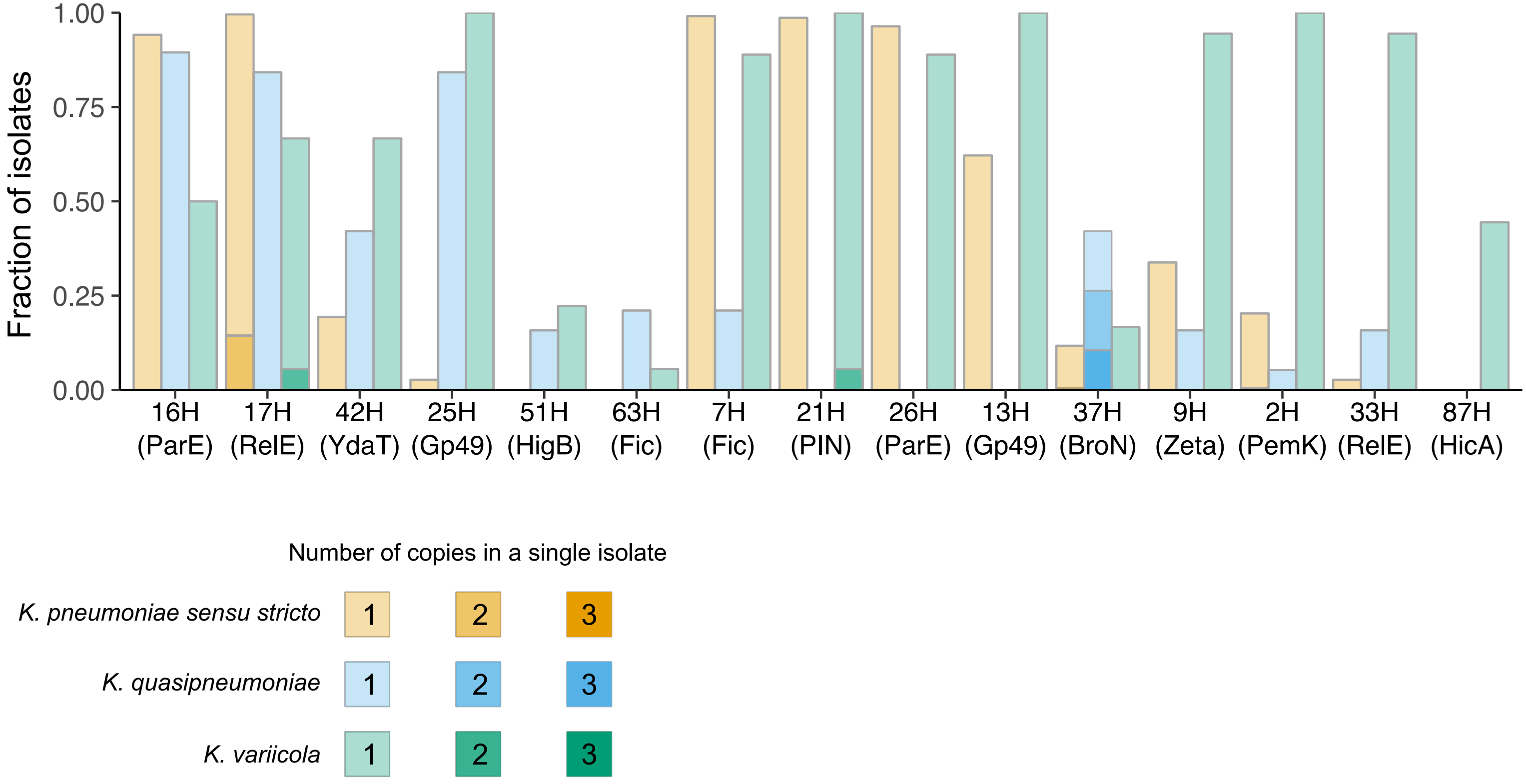

Figure S5: Fraction of isolates within each of the *K. pneumoniae* species possessing each of the toxin groups. Darker shades indicate multiple copies of the toxin.

Figure S6: Operon structures of ubiquitous toxins. Operon structures on the x-axis labelled according to SLING output (H = toxin, P = upstream and downstream antitoxins). Coloured square indicates presence of operon, white square absence. Operons grouped according by toxin. See Supplementary Table S2 for toxin label details.

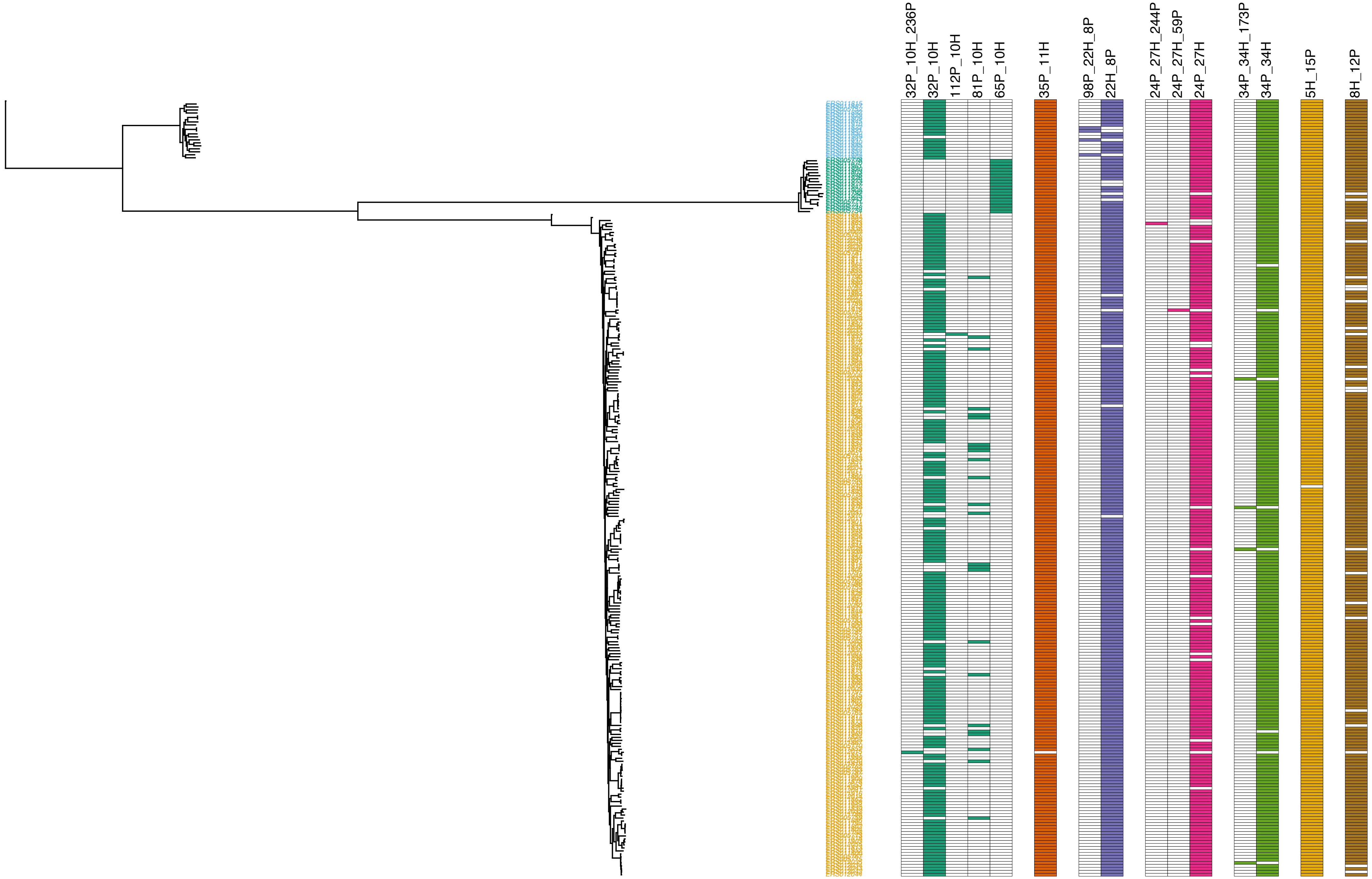

Figure S7: Operon structures of lineage associated toxins. Operon structures on the x-axis labelled according to SLING output (H = toxin, P = upstream and downstream antitoxins). Coloured square indicates presence of operon, white square absence. Operons grouped according by toxin. See Supplementary Table S2 for toxin label details.

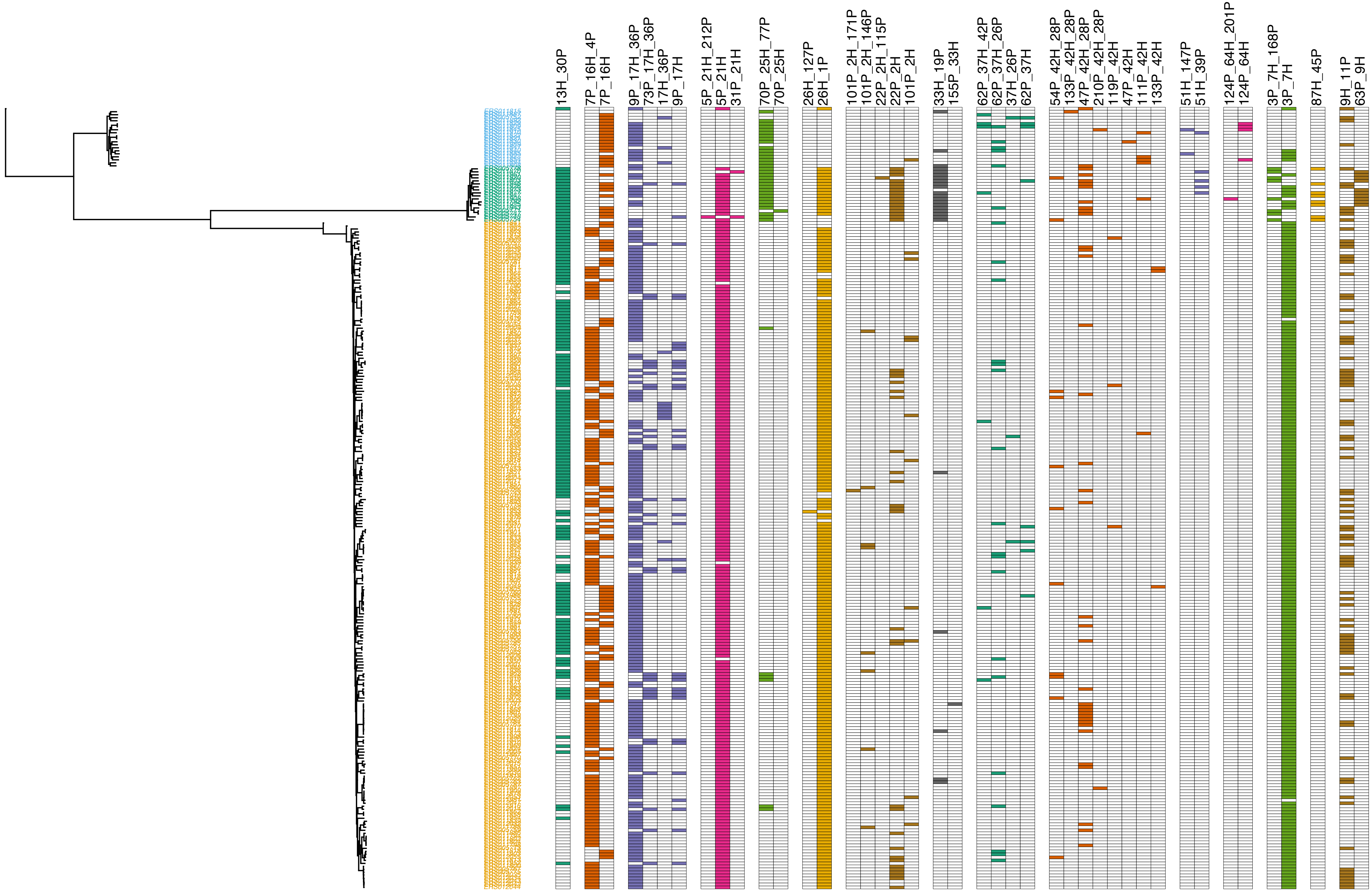

Figure S8: Operon structures of sporadically spread toxins. Operon structures on the x-axis labelled according to SLING output (H = toxin, P = upstream and downstream antitoxins). Coloured square indicates presence of operon, white square absence. Operons grouped according by toxin. See Supplementary Table S2 for toxin label details.

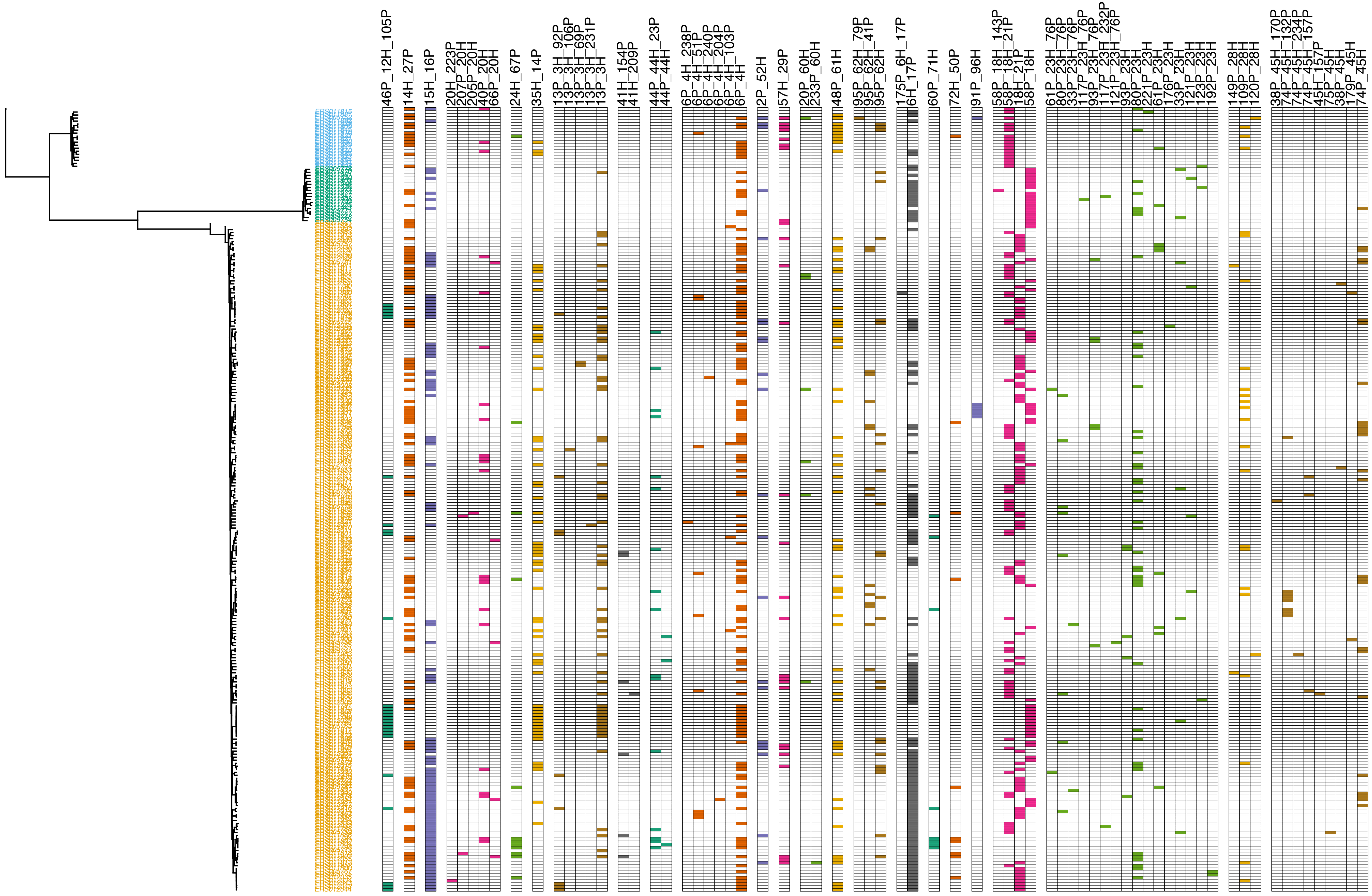

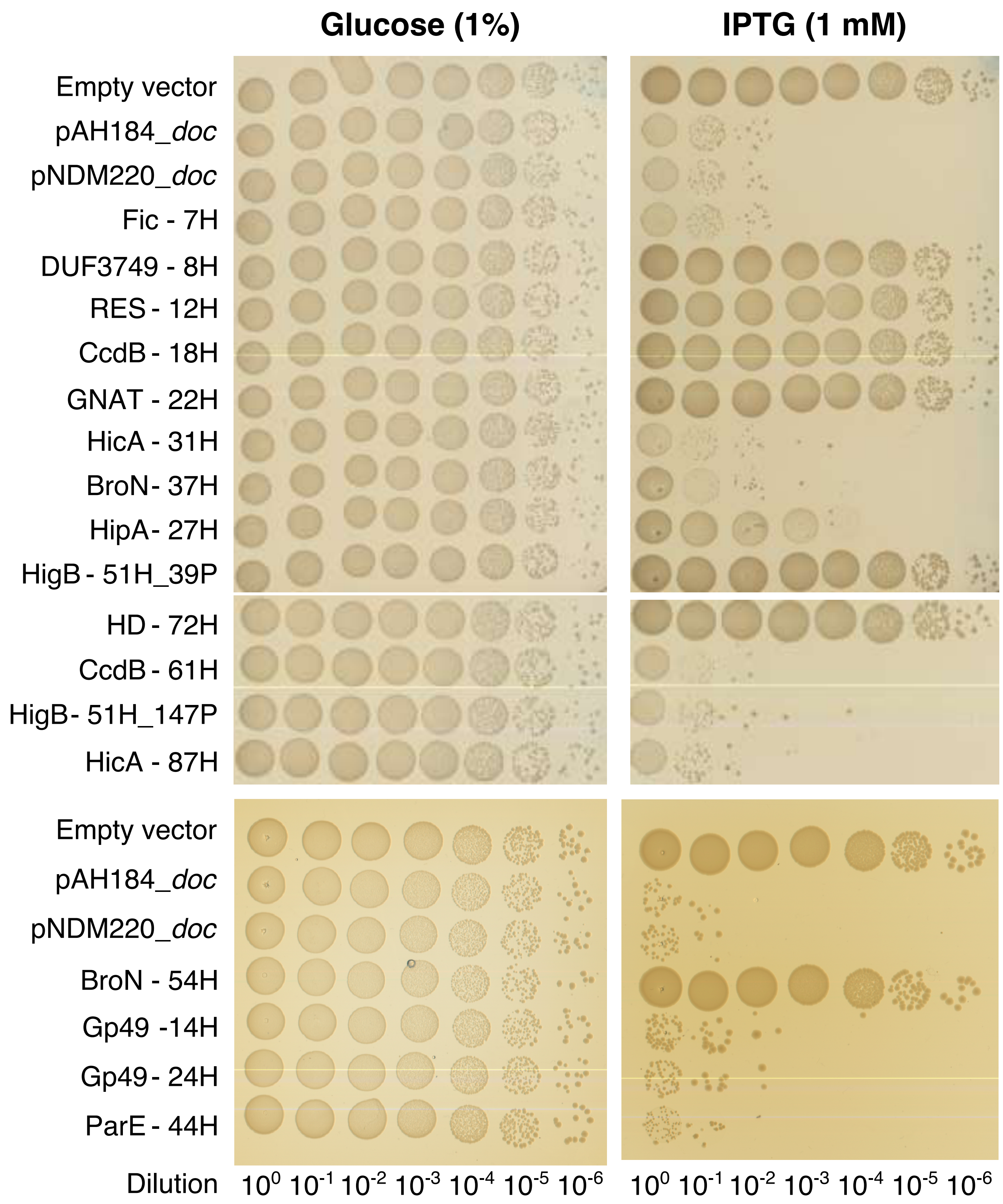

Figure S9: Phenotypic testing of selected toxins. LB agar plates were supplemented with 1mM IPTG for the induction of toxin P*lac* promoters'. Overnight cultures were serially diluted (10^{-1} to 10^{-6}) in PBS containing the inducing supplements.
